# Reservoir‐excess pressure parameters are independently associated with NT‐proBNP in older adults

**DOI:** 10.1002/ehf2.14926

**Published:** 2024-07-01

**Authors:** Kunihiko Aizawa, Alun D. Hughes, Francesco Casanova, Kim M. Gooding, Phillip E. Gates, David M. Mawson, Jennifer Williams, Isabel Goncalves, Jan Nilsson, Faisel Khan, Helen M. Colhoun, Carlo Palombo, Kim H. Parker, Angela C. Shore

**Affiliations:** ^1^ Vascular Research Centre, NIHR Exeter Clinical Research Facility University of Exeter Medical School Exeter UK; ^2^ MRC unit for Lifelong Health and Ageing, Institute of Cardiovascular Science University College London London UK; ^3^ Department of Clinical Sciences Malmö Lund University Malmö Sweden; ^4^ Department of Cardiology Skåne University Hospital Malmö Sweden; ^5^ Division of Systems Medicine University of Dundee Dundee UK; ^6^ Centre for Genomic and Experimental Medicine University of Edinburgh Edinburgh UK; ^7^ Department of Surgical, Medical, Molecular and Critical Area Pathology University of Pisa Pisa Italy; ^8^ Department of Bioengineering Imperial College London UK

**Keywords:** Ageing, Blood pressure, Biomarker, Cardiovascular disease, Left ventricle

## Abstract

**Aims:**

Parameters derived from reservoir‐excess pressure analysis have been demonstrated to predict cardiovascular events. Thus, altered reservoir‐excess pressure parameters could have a detrimental effect on highly‐perfused organs like the heart. We aimed to cross‐sectionally determine whether reservoir‐excess pressure parameters were associated with N‐terminal pro‐brain‐type natriuretic peptide (NT‐proBNP) in older adults.

**Methods:**

We studied 868 older adults with diverse cardiovascular risk. Reservoir‐excess pressure parameters were obtained through radial artery tonometry including reservoir pressure integral, peak reservoir pressure, excess pressure integral (INTXSP), systolic rate constant (SRC) and diastolic rate constant (DRC). Plasma levels of NT‐proBNP, as a biomarker of cardiac overload, were analysed by the Proximity Extension Assay technology.

**Results:**

Multivariable linear regression analyses revealed that all reservoir‐excess pressure parameters studied were associated with NT‐proBNP after adjusting for age and sex. After further adjustments for conventional cardiovascular risk factors, INTXSP [*β* = 0.191 (95% confidence interval, CI: 0.099, 0.283), *P* < 0.001], SRC [*β* = −0.080 (95% CI: −0.141, −0.019), *P* = 0.010] and DRC [*β* = 0.138 (95% CI: 0.073, 0.202), *P* < 0.001] remained associated with NT‐proBNP. Sensitivity analysis found that there were occasions where the association between SRC and NT‐proBNP was attenuated, but both INTXSP and DRC remained consistently associated with NT‐proBNP.

**Conclusions:**

The observed associations between reservoir‐excess pressure parameters and NT‐proBNP suggest that altered reservoir‐excess pressure parameters may reflect an increased load inflicted on the left ventricular cardiomyocytes and could have a potential to be utilized in the clinical setting for cardiovascular risk stratification.

## Introduction

Elevated blood pressure (BP) is the leading cause of adverse cardiovascular outcomes.^1^, ^2^ The accurate identification of cardiovascular risk associated with BP is therefore one of the important areas for an effective mitigation of cardiovascular disease (CVD). In this regard, an analysis of the BP waveform is expected to extract additional information to help clinical decision making to reduce cardiovascular risk burden. However, the conventional BP waveform analysis mainly focuses on extreme points on the waveform such as peak (systolic BP) and nadir (diastolic BP) and parameters calculated from those points. With this approach, subtle alterations in the BP waveform morphology may be overlooked and there may be residual BP‐related cardiovascular risks unquantified by conventional BP waveform analysis.^3^ This proposition calls for an alternative approach that can extract physiological and clinical information embedded in the BP waveform.

Reservoir‐excess pressure analysis provides a detailed analysis of BP waveform morphology. Not only does it identify extreme points on the BP waveform, but it also extracts physiological and clinical information contained in the waveform by separating the BP waveform into two components: (1) the reservoir pressure component that reflects the theoretical minimum hydraulic work necessary to generate a given stroke volume and (2) the excess pressure component that is an index of unnecessary work done by the left ventricle in each cardiac cycle.^4^ The parameters derived from the analysis have been demonstrated to predict cardiovascular events independently of conventional risk factors including brachial systolic BP.^5^, ^6^, ^7^, ^8^, ^9^, ^10^, ^11^, ^12^ Furthermore, we have recently shown an association between baseline reservoir pressure integral (INTPR) and the decline in estimated glomerular filtration rate (eGFR) over 3 years in older adults.^13^ These observations indicate that alterations in reservoir‐excess pressure parameters could have a detrimental effect on target organs such as the heart. However, because reservoir‐excess pressure analysis is still in its early years, the evidence showing the association between reservoir‐excess pressure parameters and biomarkers of cardiac damage is limited.^6^, ^14^

N‐terminal pro‐B‐type natriuretic peptide (NT‐proBNP), an established biomarker of cardiac overload, is synthesized mainly by cardiomyocytes in response to stretch.^15^ The concentration of NT‐proBNP is increased in the presence of left ventricular dysfunction,^16^, ^17^ and it possesses prognostic information independent of conventional risk factors.^16^ This raises a possibility that an altered BP waveform morphology would reflect NT‐proBNP concentration, and reservoir‐excess pressure analysis could detect those changes from BP waveform morphology. Therefore, we aimed to cross‐sectionally determine in this study whether reservoir‐excess pressure parameters were associated with NT‐proBNP in older adults.

## Methods

### Participants

Participants were recruited from Dundee and Exeter (both United Kingdom) and Malmö (Sweden) for the SUrrogate markers for Micro‐ and Macrovascular hard endpoints for Innovative diabetes Tools‐Vascular Imaging Prediction (SUMMIT‐VIP) study. Data collected from participants who had raw radial pressure waveform data as well as NT‐proBNP data at baseline (*n* = 868) were used for this analysis. The details of the main study including the criteria for inclusion/exclusion have been described elsewhere.^18^, ^19^ Demographic and clinical characteristics data including physical and laboratory analyses were obtained based on the predefined main study protocol at each site. All study procedures were complied with the Declaration of Helsinki, and approved by the UK National Research Ethics Service South West Committee, East of Scotland Research Ethics Service and the institutional ethics committee at the Lund University, Sweden. Written informed consent was obtained from all participants.

### Acquisition of radial pressure waveform and derivation of reservoir‐excess pressure parameters

**Figure 1:**
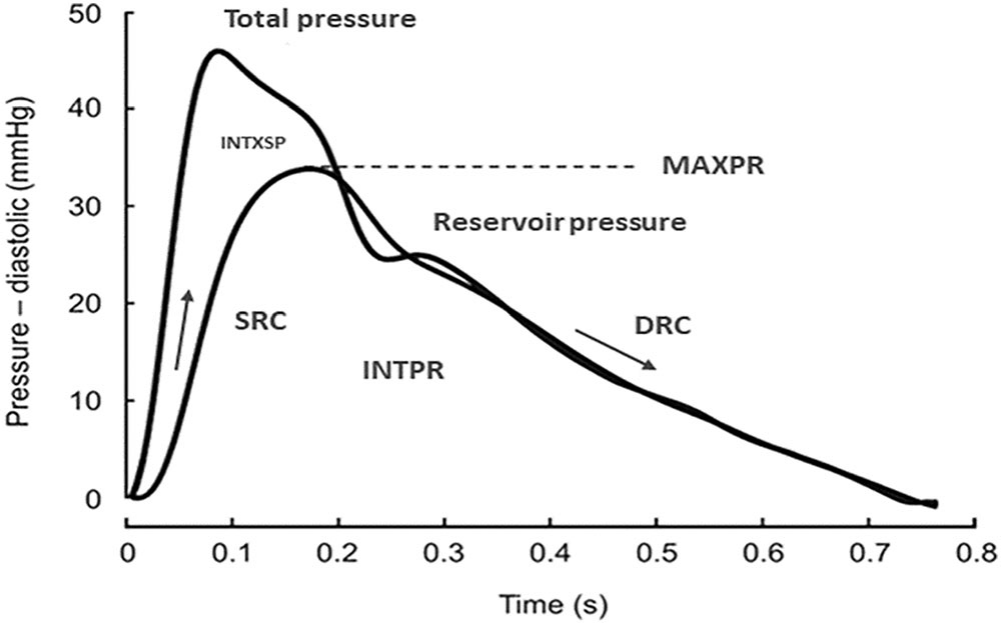
A schematic representation of reservoir‐excess pressure separation in the radial artery.^12^ Total pressure is the acquired radial pressure waveform and reservoir pressure is the calculated waveform. DRC, diastolic rate constant; INTPR, reservoir pressure integral; INTXSP, excess pressure integral; MAXPR, peak reservoir pressure; SRC, systolic rate constant.

The details of our radial pressure waveform acquisition method as well as derivation of reservoir‐excess pressure parameters have been described elsewhere.^12^, ^13^ Briefly, right radial artery pressure waveforms were recorded with a high‐fidelity micromanometer attached to a SphygmoCor system (Version 8.2, AtCor Medical Pty Ltd, West Ryde, New South Wales, Australia) over 10 s after the participants lying supine on an examining bed, having rested for 10 min before the assessment. Dedicated inbuilt software then processed acquired waveforms to calculate an ensemble‐averaged radial pressure waveform calibrated by brachial systolic and diastolic BP (as per the manufacturer's recommendation) using a validated semi‐automated oscillometric device (Omron M6, Hoofddorp, the Netherlands). The ensemble‐averaged radial pressure waveform was then used to calculate reservoir‐excess pressure parameters based on the pressure‐alone approach. A review of the method that includes its theoretical basis and validation has been published recently.^4^ In the reservoir‐excess pressure analysis, the measured pressure waveform can be separated into (1) a reservoir pressure component, which varies in magnitude through changes in the resistance to outflow from the reservoir, the reservoir compliance and the asymptotic pressure^20^ and (2) an excess pressure component which is the difference between the measured pressure waveform and reservoir pressure. The calculation of the reservoir pressure depends on determination of two rate constants: the systolic rate constant (SRC) which is the inverse of the product of the constant of proportionality between the excess pressure and the arterial inflow and the total arterial compliance; and DRC which is the inverse of the product of the peripheral vascular resistance and the total arterial compliance. Reservoir‐excess pressure parameters analysed in this study were (1) reservoir pressure integral (INTPR), (2) peak reservoir pressure (MAXPR), (3) excess pressure integral (INTXSP), (4) SRC and (5) DRC. A schematic example of the reservoir‐excess pressure separation is shown in *Figure* 1.

### Cardiovascular risk factors and NT‐proBNP

Height, body weight and body mass index were obtained using a standardized protocol. Total and HDL cholesterol and HbA1c were measured from participants' fasting blood samples in each study centre. eGFR was calculated using the Chronic Kidney Disease Epidemiology Collaboration creatinine equation.^21^

NT‐proBNP was measured as a biomarker of myocardial stress using the Proseek Multiplex ${\mathrm{CVD}}^{96\times 96}$ reagents kit (Olink Bioscience, Uppsala, Sweden) at the Clinical Biomarkers Facility, Science for Life Laboratory, Uppsala, Sweden, as described previously.^19^, ^22^ All samples were analysed in the same run. Data analysis was performed by a pre‐processing normalization procedure using Olink Wizard for GenEx (Multid Analyses, Gothenburg, Sweden). Values are presented in a log2 scale. Data regarding intra‐ and inter‐assay variations as well as general calibrator curves to calculate the approximate concentrations are available on the OLINK home page (http://www.olink.com).

### Statistical analysis

Data are presented as means ± *SD*, median (interquartile range), or number (%). Skewed data were appropriately transformed for statistical analysis. Univariable and multivariable linear regression analyses were performed to quantify associations between reservoir‐excess pressure parameters and NT‐proBNP. For multivariable linear regression analyses, the following variables were considered to be potential confounders: age, sex, total and HDL cholesterol, current smoking, presence of type 2 diabetes, brachial systolic BP, pharmacological anti‐hypertensive treatment, study centre, body mass index, previous history of CVD, baseline eGFR and resting heart rate (assigned as above/below median due to collinearity as a continuous variable). Reservoir‐excess pressure parameters were standardized before entering into the multivariable linear regression analysis to allow comparisons across the parameters (i.e., 1‐standard deviation increase). A sensitivity analysis was performed by replacing/adding a potential covariate in multivariable linear regression models. Unlike the main analysis, the number of participants entered into the sensitivity analysis varied due to missing covariate data and thus was specified in each covariate entered in the model (Tables S1–S6). Results were summarized as *β* [95% confidence intervals (CI)]. Statistical analysis was conducted using IBM SPSS Statistics 28 (IBM, Armonk, NY) and statistical significance was set at *P* < 0.05 (two sided).

## Results

Selected characteristics, reservoir‐excess pressure parameters and NT‐proBNP levels of the study participants included in this analysis are presented in *Table* 1. Participants were on average ~70 years old and overweight, and the majority were receiving anti‐hypertensive medications. Their systolic and diastolic BP was adequately controlled. More than 60% and 50% of the participants had type 2 diabetes and CVD, respectively.

**Table 1:** Selected characteristics, reservoir‐excess pressure parameters and NT‐proBNP of the study participants.

	Values
Age, years	69.5 ± 8.0
Female, *n* (%)	319 (36.8)
Body mass index, kg/m^2^	28.5 (25.4–32.2)
Total CHOL, mmol/L	4.1 (3.6–4.9)
HDL CHOL, mmol/L	1.3 (1.1–1.6)
HbA1c, mmol/mol	47 (40–58)
Systolic BP, mmHg	136 ± 18
Diastolic BP, mmHg	75 ± 9
Heart rate, bpm	60 ± 10
eGFR, mL/min/1.73m^2^	78.8 ± 26.7
Current smoking, *n* (%)	78 (9.0)
Type 2 diabetes, *n* (%)	534 (61.6)
Cardiovascular disease, *n* (%)	434 (50.0)
Acute myocardial infarction, *n* (%)	215 (24.8)
Unstable angina, *n* (%)	122 (14.1)
Atrial fibrillation, *n* (%)	39 (4.5)
Coronary artery bypass graft, *n* (%)	147 (16.9)
Percutaneous coronary intervention, *n* (%)	155 (17.9)
Stroke, *n* (%)	51 (5.9)
Transient ischaemic attacks, *n* (%)	63 (7.3)
HTRx, *n* (%)	637 (73.5)
Statin treatment, n (%)	595 (68.6)
INTPR, mmHg·s	91.4 ± 17.0
MAXPR, mmHg	107.7 ± 14.5
INTXSP, mmHg·s	7.4 (5.9–9.3)
SRC, 1/s	6.8 (5.7–7.9)
DRC, 1/s	2.4 (1.9–2.9)
NT‐proBNP, au	21.1 (12.0–35.0)

*Note*: Data are presented as means ± *SD*, median (interquartile ranges) or number (%).

Abbreviations: BP, blood pressure; CHOL, cholesterol; DRC, diastolic rate constant; eGFR, estimated glomerular filtration rate; HbA1c, haemoglobin A1c; HTRx, pharmacological hypertensive treatment; INTPR, reservoir pressure integral; INTXSP, excess pressure integral; MAXPR, peak reservoir pressure; NT‐proBNP, N‐terminal pro‐B‐type natriuretic peptide; SRC, systolic rate constant.

**Figure 2:**
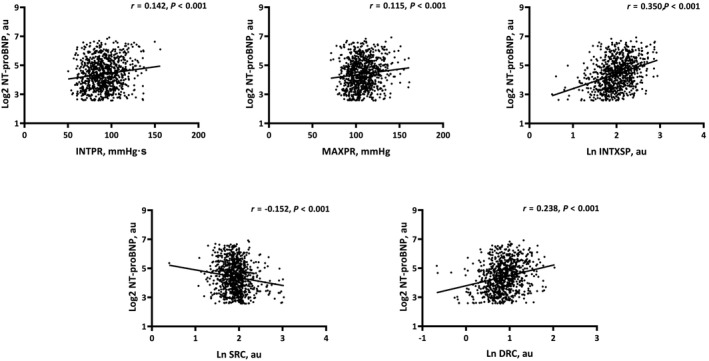
Univariable associations between reservoir‐excess pressure parameters and NT‐proBNP. DRC, diastolic rate constant; LN, natural logarithm; INTPR, reservoir pressure integral; INTXSP, excess pressure integral; MAXPR, peak reservoir pressure; SRC, systolic rate constant; NT‐proBNP, N‐terminal pro‐B‐type natriuretic peptide.

*Figure* 2 shows univariable associations between reservoir‐excess pressure parameters and NT‐proBNP. INTPR, MAXPR, INTXSP and DRC were all positively associated with NT‐proBNP (all *P* < 0.001) whereas SRC was inversely associated with NT‐proBNP (*P* < 0.001).

Multivariable linear regression analyses were performed to determine independent associations between reservoir‐excess pressure parameters and NT‐proBNP. As can be seen in *Table* 2, all reservoir‐excess pressure parameters were associated with NT‐proBNP after adjusting for age and sex (Model 1). Further adjustments for additional cardiovascular risk factors and study centre confirmed that INTXSP, SRC and DRC were associated with NT‐proBNP independently of conventional cardiovascular risk factors (Model 2). Of note, when the product of INTXSP and heart rate (INTXSP*HR: i.e., excess cardiac workload per minute) was included in the analyses instead of INTXSP, INTXSP*HR was associated with NT‐proBNP after adjusting for age and sex [Model‐1; *β* = 0.184 (95% CI: 0.114, 0.253), *P* < 0.001]. The association remained, although attenuated, after further adjustments for additional cardiovascular risk factors and study centres [Model‐2; *β* = 0.093 (95% CI: 0.003, 0.183), *P* = 0.043].

**Table 2:** Multivariable linear regression analyses to determine associations between reservoir‐excess pressure parameters and NT‐proBNP independent of risk factors.

	Model 1: *β* (95% CI)	Model 2: *β* (95% CI)
INTPR	0.132 (0.068, 0.195), *P* < 0.001	0.082 (−0.011, 0.174), *P* = 0.084
MAXPR^a^	0.065 (0.000, 0.129), *P* = 0.049	−0.076 (−0.182, 0.031), *P* = 0.163
INTXSP	0.272 (0.204, 0.340), *P* < 0.001	0.191 (0.099, 0.283), *P* < 0.001
SRC	−0.119 (−0.183, −0.055), *P* < 0.001	−0.080 (−0.141, −0.019), *P* = 0.010
DRC	0.172 (0.107, 0.236), *P* < 0.001	0.138 (0.073, 0.202), *P* < 0.001

*Note*: Data are presented as *β* (95% confidence intervals). Model 1 includes age and sex. Model 2 includes total and HDL cholesterol, current smoking, presence of type 2 diabetes, brachial systolic BP, pharmacological anti‐hypertensive treatment, study centre, body mass index, history of cardiovascular disease, estimated glomerular filtration rate and heart rate (above/below median) in addition to Model 1.

Abbreviations: *β*, beta; CI, confidence intervals; DRC, diastolic rate constant; INTPR, reservoir pressure integral; INTXSP, excess pressure integral; MAXPR, peak reservoir pressure; SRC, systolic rate constant.

^a^ The extent of collinearity was *r* = 0.797 between MAXPR and brachial systolic BP, and variance inflation factor (VIF) for MAXPR was 3.235 and for brachial systolic BP was 3.297.

A series of sensitivity analyses was then performed. The first analysis was to replace brachial systolic BP with aortic systolic BP/aortic PP/brachial PP in the Model 2, to add aortic augmentation index corrected at heart rate 75 bpm/carotid‐femoral pulse wave velocity into the Model 2, and to replace the pharmacological anti‐hypertensive treatment with the use of renin‐angiotensin system antagonists in the Model 2 (*Table* S1). The strength of associations observed in the main analyses between INTXSP, SRC, DRC and NT‐proBNP mostly remained in these analyses, although there were a few occasions where the association between SRC and NT‐proBNP was attenuated with those additional analyses. The second analysis was to replace brachial systolic BP with brachial diastolic BP/mean arterial pressure in the Model 2 (Table S2). On both occasions, all reservoir‐excess pressure parameters except MAXPR with mean arterial pressure were associated with NT‐proBNP. The third analysis was to add reservoir‐excess pressure parameters and aortic systolic BP/aortic PP in Model 2 after each parameter was standardized (Table S3). When each reservoir‐excess pressure parameter and aortic systolic BP were entered into the Model 2 together, INTXSP (but not aortic systolic BP) was independently associated with NT‐proBNP whereas SRC and DRC were independently associated with NT‐proBNP in addition to aortic systolic BP. Furthermore, when each reservoir‐excess pressure parameter and aortic PP were entered into the Model 2 together, DRC was independently associated with NT‐proBNP in addition to aortic PP. Additionally, aortic PP (but not INTPR, MAXPR or SRC) was independently associated with NT‐proBNP whereas INTXSP and aortic PP showed collinearity that prevented isolating the influence of each haemodynamic parameter to NT‐proBNP. The fourth analysis was to determine the effect of sex on the associations (Table S4). Similar to what we found in the main analysis, INTXSP and DRC were associated with NT‐proBNP in both sexes. In addition, the magnitude of associations between INTXSP, DRC and NT‐proBNP was found to be similar in both sexes with no statistically significant sex * reservoir‐excess pressure parameter interaction term. The fifth analysis was to determine the effect of age (stratified by below/above 65 years) on the associations (Table S5). INTXSP and DRC were associated with NT‐proBNP in both age groups independently of conventional cardiovascular risk factors and other relevant covariates. In addition, the magnitude of associations between INTXSP, DRC and NT‐proBNP was found to be similar in both age groups with no statistically significant age * reservoir‐excess pressure parameters interaction term. The sixth analysis was to determine the effect of CVD (stratified as the presence/absence of CVD) on the associations (Table S6). INTXSP and DRC were associated with NT‐proBNP in both groups independently of conventional cardiovascular risk factors and other relevant covariates, although slightly less so in the Model 2 in those without CVD. SRC was also independently associated with NT‐proBNP in those with CVD. Additionally, there was no observation of statistically significant CVD * reservoir‐excess pressure parameters interaction term. The final analysis was to add both SRC and DRC into the same multivariable linear regression model together (Model 2). SRC [*β* = −0.136 (−0.204, −0.067), *P* < 0.001] and DRC [*β* = 0.180 (0.112, 0.247), *P* < 0.001] were found to be both independently associated with NTproBNP in this model. Similar findings were also observed when both INTXSP and DRC were forced into the same multivariable linear regression model (Model 2). INTXSP [*β* = −0.189 (0.096, 0.282), *P* < 0.001] and DRC [*β* = 0.139 (0.075, 0.203), *P* < 0.001] were found to be both independently associated with NT‐proBNP.

## Discussion

We demonstrate that reservoir‐excess pressure parameters, specifically INTXSP, SRC and DRC, were associated with NT‐proBNP in older adults with diverse cardiovascular risk. The observed associations were independent of traditional cardiovascular risk factors including brachial systolic BP as well as other haemodynamic parameters such as aortic systolic BP, PP and carotid‐femoral pulse wave velocity. Of note, there were occasions where the strength of association between SRC and NT‐proBNP was attenuated in the sensitivity analyses, but both INTXSP and DRC remained consistently associated with NT‐proBNP. These findings, observed for the first time to our knowledge, suggest that altered reservoir‐excess pressure parameters could be a marker of increased myocardial stress, and may reflect subclinical deterioration of left ventricular structure and function.

The prognostic utility of reservoir‐excess pressure parameters for the prediction of cardiovascular events has already been demonstrated in various populations,^5^, ^6^, ^7^, ^8^, ^9^, ^10^, ^11^, ^12^ suggesting that alterations in reservoir‐excess pressure parameters could indicate a detrimental effect on highly perfused organs such as the brain, heart and kidneys. Indeed, the previously observed cross‐sectional association of reservoir‐excess pressure parameters with brain structure^23^ and renal function^24^ supports this contention. Furthermore, we and others have shown that there was an association between baseline INTPR in people with type 2 diabetes^13^ as well as a change in INTXSP in healthy older adults,^25^ respectively, and a decline in eGFR at follow‐up. The former association was independent of traditional cardiovascular risk factors including brachial systolic BP as well as conventional haemodynamic parameters such as aortic systolic BP and carotid‐femoral pulse wave velocity.^13^ The association of reservoir‐excess pressure parameters with NT‐proBNP observed in this study adds to a growing body of evidence demonstrating that reservoir‐excess pressure analysis has a capability to extract physiological and clinical information of target organ damage from the BP waveform morphology that may have been overlooked by conventional BP waveform analysis.

The reservoir pressure component makes a major contribution to the diastolic phase of the BP waveform while the excess pressure component mainly contributes to the early systolic phase of BP waveform.^26^ Thus, it is intriguing that reservoir‐excess pressure parameters reflecting both the systolic (INTXSP and SRC) and diastolic (DRC) phases of the BP waveform play a role in the observed associations with NT‐proBNP.

### Excess pressure integral

Excess pressure, defined as the difference between total measured pressure and reservoir pressure, is proposed to represent superfluous work that the left ventricle has to perform in each heartbeat to eject a stroke volume,^26^ indicating an inefficient ventricular–vascular interaction. As previously alluded to,^27^ an accumulation of excess haemodynamic load over time can adversely affect the left ventricle structurally and functionally and, when sustained, may lead to a structural maladaptation (e.g., left ventricular hypertrophy)^6^, ^14^ and a subsequent development of cardiovascular events.^6^ Although speculative due to our cross‐sectional study design, the association of INTXSP with NT‐proBNP could be attributable to increased left ventricular stiffness, elevated systolic wall stress and/or structural maladaptation by sustained excess haemodynamic load.^28^ Therefore, the positive association observed between INTXSP and NT‐proBNP in this study provides additional support for the premise that INTXSP is a novel measure of circulatory dysfunction^6^ and may provide an additional estimate of left ventricular overload. We acknowledge that INTXSP was calculated from the radial BP waveform in this study, and hence, there is information embedded that is relevant to local (i.e., radial artery) haemodynamic conditions in addition to the information on ventricular–vascular interaction.^26^ Given the consistent association observed between INTXSP and NT‐proBNP in multivariable linear regression analyses, however, we think it likely that the information on ventricular–vascular interaction is preserved in INTXSP rather than being masked by the local haemodynamic condition.

### Systolic rate constant

Contrary to INTXSP and DRC, we observed an inverse association between SRC and NT‐proBNP in this study, meaning that higher SRC values reflect a better left ventricular ejection functioning. SRC is the rate at which reservoir pressure increases (i.e., reservoir filling) during systole,^26^ and is inversely proportional to the product of total arterial compliance and an impedance that probably equates aortic characteristic impedance. In light of this, a higher SRC may indicate lower values of large elastic artery stiffness or lower values of aortic characteristic impedance, which opposes a faster rate of reservoir filling. Furthermore, the geometry of the aorta and other large elastic arteries may also play a role in SRC through its influence on large elastic artery stiffness and characteristic impedance. Changes in proximal aortic diameter, in particular, have a powerful effect on aortic characteristic impedance.^29^ A protective association of higher SRC with cardiovascular events has been demonstrated in elderly hypertensives^7^ and may explain pathophysiological consequences of lower SRC on the left ventricle.

### Diastolic rate constant

It is noteworthy that we found a positive association between DRC and NT‐proBNP in this study. DRC measures the rate of reservoir pressure reduction during diastole,^26^ that is, it reflects how fast the reservoir is emptying. Viewed with reference to a simple Windkessel model, DRC is inversely proportional to the product of total arterial compliance and peripheral vascular resistance. It is challenging to tease out individual contributions of each parameter to the greater DRC; however, because a greater large elastic artery stiffness is a well‐known observation in older adults and in people with various cardiovascular risks (i.e., a majority of our study cohort), it is plausible that reduced total arterial compliance may play a pivotal role in the observed association between DRC and NT‐proBNP. With large elastic artery stiffening, the buffering function that accommodates the intermittent blood flow ejected from the left ventricle during systole is compromised, increasing the haemodynamic load. This is also consistent with our explanations for the associations of INTXSP and SRC with NT‐proBNP observed in this study. However, increased arterial stiffness and an increased rate of decline in diastolic pressure also reduce coronary artery perfusion, which may predispose to subendocardial ischaemia.^30^ The reduced coronary artery perfusion could then lead to myocardial damage and adverse cardiac remodelling,^31^ which may make the heart more vulnerable to the loads imposed by INTXSP and SRC. The observed association between DRC and NT‐proBNP in this study may imply the existence of a potential vicious cycle that aggravates left ventricular structure and function over time and increases cardiovascular risks in older adults.

We acknowledge that there are limitations in this study. Our study cohort comprised older adults with diverse cardiovascular risk profiles. Therefore, the findings from this study may not be generalizable to other patient cohorts, such as people with hypertension or with type 2 diabetes. In addition, due to the cross‐sectional design of this study, we are unable to infer a causal relationship between reservoir‐excess pressure parameters and NT‐proBNP. Finally, echocardiography measures such as left ventricular structure and function, other cardiac‐related biomarkers such as cardiac troponins or some of the medical history associated with heart failure such as valvular disease were not available in this study; these could have helped interpret our findings.

## Conclusions

This study demonstrates that reservoir‐excess pressure parameters were associated with a biomarker of myocardial stress, NT‐proBNP, in older adults with diverse cardiovascular risk, independently of established cardiovascular risk factors and conventional haemodynamic parameters. The observed associations indicate that altered reservoir‐excess pressure parameters may reflect an increased stress inflicted on the left ventricular cardiomyocytes and may help identify time‐varying pathophysiological mechanisms that contribute to left ventricular overload in the clinical setting, adding to those derived from conventional BP waveform analysis.

## Funding

This study was supported by the European Union's Seventh Framework Programme (FP7/2007–2013) for the Innovative Medicines Initiative under grant agreement number IMI/115006 (the SUMMIT consortium) and in part by the National Institute of Health and Care Research (NIHR) Exeter Clinical Research Facility. The views expressed are those of the authors and not necessarily those of the UK National Health Service, the NIHR or the UK Department of Health and Social Care. A. D. H. receives support from the British Heart Foundation, the Economic and Social Research Council (ESRC), the Horizon 2020 Framework Programme of the European Union, the National Institute on Aging, the National Institute for Health and Care Research University College London Hospitals Biomedical Research Centre, the UK Medical Research Council and works in a unit that receives support from the UK Medical Research Council. I. G. was supported by Swedish Research Council, Swedish Heart and Lung Foundation, Skåne University Hospital Foundations and the Lund University Diabetes Center–Industrial Research Center from the Swedish Foundation of Strategic Research Dnr IRC15‐0067 and the Strategic Research Area Exodiab, Dnr 2009–1039.

## Conflict of interest

None.

## Supporting information


**Table S1.** Additional multivariable linear regression analyses to determine the associations between reservoir‐excess pressure parameters and NT‐proBNP when (A) other haemodynamic measures aortic systolic BP, aortic PP, brachial PP, aortic augmentation index, carotid‐femoral pulse wave velocity were forced into models in place of brachial systolic BP; (B) any anti‐hypertensive treatments was replaced with RAS antagonists use.
**Table S2.** Additional multivariable linear regression analyses to determine independent associations between reservoir‐excess pressure parameters and NT‐proBNP by replacing brachial diastolic BP and mean arterial pressure.
**Table S3.** Additional multivariable linear regression analyses to determine independent associations between reservoir‐excess pressure parameters, aortic parameters and NT‐proBNP.
**Table S4.** Additional multivariable linear regression analyses to determine independent associations between reservoir‐excess pressure parameters and NT‐proBNP by stratifying participants' sex.
**Table S5.** Additional multivariable linear regression analyses to determine independent associations between reservoir‐excess pressure parameters and NT‐proBNP by stratifying participants above/below 65 years of age.
**Table S6.** Additional multivariable linear regression analyses to determine independent associations between reservoir‐excess pressure parameters and NT‐proBNP by stratifying participants with/without the history of cardiovascular disease.
