# Supplementary material for: Reservoir‐excess pressure parameters are independently associated with NT‐proBNP in older adults
Source: ESC Heart Fail. 2024 Jul 1;11(5):3290–8. doi: 10.1002/ehf2.14926 (PMC11424359; doi:10.1002/ehf2.14926)
Supplement: Supplementary file 1 — Table S1. Additional multivariable linear regression analyses to determine the associations between reservoir‐excess pressure parameters and NT‐proBNP when (A) other haemodynamic measures aortic systolic BP, aortic PP, brachial PP, aortic augmentation index, carotid‐femoral pulse wave velocity were forced into models in place of brachial systolic BP; (B) any anti‐hypertensive treatments was replaced with RAS antagonists use. Table S2. Additional multivariable linear regression analyses to determine independent associations between reservoir‐excess pressure parameters and NT‐proBNP by replacing brachial diastolic BP and mean arterial pressure. Table S3. Additional multivariable linear regression analyses to determine independent associations between reservoir‐excess pressure parameters, aortic parameters and NT‐proBNP. Table S4. Additional multivariable linear regression analyses to determine independent associations between reservoir‐excess pressure parameters and NT‐proBNP by stratifying participants' sex. Table S5. Additional multivariable linear regression analyses to determine independent associations between reservoir‐excess pressure parameters and NT‐proBNP by stratifying participants above/below 65 years of age. Table S6. Additional multivariable linear regression analyses to determine independent associations between reservoir‐excess pressure parameters and NT‐proBNP by stratifying participants with/without the history of cardiovascular disease. [file EHF2-11-3290-s001.docx]

**SUPPLEMENTAL MATERIAL**

**Reservoir-Excess Pressure Parameters are Independently Associated with NT-proBNP in Older Adults**

***Authors:***

Kunihiko Aizawa,^1^ Alun D Hughes,^2^ Francesco Casanova,^1^, Kim M Gooding,^1^ Phillip E Gates,^1^ David M Mawson,^1^ Jennifer Williams,^1^ Isabel Goncalves,^3,4^ Jan Nilsson,^3^ Faisel Khan,^5^ Helen M Colhoun,^6^ Carlo Palombo,^7^ Kim H Parker,^8^ Angela C Shore.^1^

***Affiliations:***

^1^Diabetes and Vascular Medicine Research Centre, NIHR Exeter Clinical Research Facility, University of Exeter Medical School, Exeter, UK. ^2^MRC unit for Lifelong Health & Ageing, Institute of Cardiovascular Science, University College London, London, UK. ^3^Department of Clinical Sciences Malmö, Lund University, Malmö, Sweden. ^4^Department of Cardiology, Skåne University Hospital, Malmö, Sweden. ^5^Division of Systems Medicine, University of Dundee, Dundee, UK. ^6^Centre for Genomic and Experimental Medicine, University of Edinburgh, Edinburgh, UK. ^7^Department of Surgical, Medical, Molecular and Critical Area Pathology, University of Pisa, Pisa, Italy. ^8^Department of Bioengineering, Imperial College, London, UK.

***Short Title:***

Reservoir pressure and cardiac damage

***Corresponding author:***

Kunihiko Aizawa, PhD

Diabetes and Vascular Medicine Research Centre

University of Exeter Medical School

NIHR Exeter Clinical Research Facility

Barrack Road, Exeter

EX2 5AX, UK

+44 1392 403081 (TEL)

+44 1392 403027 (FAX)

k.aizawa@exeter.ac.uk

**Supplemental Table S1.** Additional multivariable linear regression analyses to determine the associations between reservoir-excess pressure parameters and NT-proBNP when A) other haemodynamic measures (aortic systolic BP, aortic PP, brachial PP, aortic augmentation index, carotid-femoral pulse wave velocity were forced into models in place of brachial systolic BP; B) any anti-hypertensive treatments was replaced with RAS antagonists use.

|  | **n** | **Model 1: β (95% CI)** | **Model 2: β (95% CI)** |
| --- | --- | --- | --- |
| ***A*** | | | |
| ***Replacing brachial systolic BP with aortic systolic BP*** | | | |
| INTPR | 852 | 0.128 (0.063, 0.192), *p*<0.001 | 0.053 (-0.054, 0.160), *p*=0.330 |
| MAXPR^a^ | 852 | 0.062 (-0.003, 0.128), *p*=0.061 | -0.295 (-0.452, -0.138), *p*<0.001 |
| INTXSP | 852 | 0.269 (0.200, 0.338), *p*<0.001 | 0.181 (0.083, 0.279), *p*<0.001 |
| SRC | 852 | -0.119 (-0.184, -0.054), *p*<0.001 | -0.079 (-0.141, -0.017), *p*=0.012 |
| DRC | 852 | 0.173 (0.108, 0.239), *p*<0.001 | 0.137 (0.072, 0.202), *p*<0.001 |
| ***Replacing brachial systolic BP with aortic PP*** | | | |
| INTPR | 852 | 0.128 (0.063, 0.192), *p*<0.001 | 0.073 (-0.014, 0.161), *p*=0.101 |
| MAXPR | 852 | 0.062 (-0.003, 0.128), *p*=0.061 | -0.034 (-0.119, 0.051), *p*=0.436 |
| INTXSP^b^ | 852 | 0.269 (0.200, 0.338), *p*<0.001 | 0.135 (-0.014, 0.284), *p*=0.075 |
| SRC | 852 | -0.119 (-0.184, -0.054), *p*<0.001 | -0.057 (-0.119, 0.005), *p*=0.072 |
| DRC | 852 | 0.173 (0.108, 0.239), *p*<0.001 | 0.125 (0.060, 0.191), *p*<0.001 |
| ***Replacing brachial systolic BP with brachial PP*** | | | |
| INTPR | 866 | 0.132 (0.068, 0.195), p<0.001 | 0.107 (0.026, 0.188), *p*=0.010 |
| MAXPR | 866 | 0.064 (-0.001, 0.129), *p*=0.052 | 0.019 (-0.056, 0.094), *p*=0.615 |
| INTXSP^c^ | 866 | 0.271 (0.203, 0.340), *p*<0.001 | 0.181 (0.071, 0.290), *p*=0.001 |
| SRC | 866 | -0.119 (-0.183, -0.055), *p*<0.001 | -0.059 (-0.120, 0.002), *p*=0.057 |
| DRC | 866 | 0.171 (0.106, 0.235), *p*<0.001 | 0.136 (0.072, 0.200), *p*<0.001 |
| ***Adding aortic augmentation index corrected at heart rate 75 bpm*** | | | |
| INTPR | 847 | 0.112 (0.046, 0.177), *p*<0.001 | 0.045 (-0.051, 0.141), *p*=0.362 |
| MAXPR^d^ | 847 | 0.061 (-0.005, 0.126), *p*=0.069 | -0.137 (-0.251, -0.023), *p*=0.018 |
| INTXSP | 847 | 0.265 (0.195, 0.334), *p*<0.001 | 0.172 (0.078, 0.266), *p*<0.001 |
| SRC | 847 | -0.116 (-0.180, -0.051), *p*<0.001 | -0.076 (-0.138, -0.015), *p*=0.015 |
| DRC | 847 | 0.178 (0.113, 0.243), *p*<0.001 | 0.134 (0.068, 0.200), *p*<0.001 |
| ***Adding carotid-femoral pulse wave velocity*** | | | |
| INTPR | 715 | 0.139 (0.070, 0.207), *p*<0.001 | 0.093 (-0.009, 0.196), *p*=0.073 |
| MAXPR^e^ | 715 | 0.060 (-0.011, 0.130), *p*=0.096 | -0.066 (-0.184, 0.053), *p*=0.276 |
| INTXSP | 715 | 0.269 (0.193, 0.346), *p*<0.001 | 0.175 (0.072, 0.278), *p*<0.001 |
| SRC | 715 | -0.108 (-0.175, -0.042), *p*=0.001 | -0.067 (-0.131, -0.003), *p*=0.039 |
| DRC | 715 | 0.163 (0.092, 0.234), *p*<0.001 | 0.139 (0.067, 0.212), *p*<0.001 |
| ***B*** | | | |
| ***Replacing the anti-hypertensive treatment with the RAS antagonists use*** | | | |
| INTPR | 860 | 0.135 (0.072, 0.199), *p*<0.001 | 0.080 (-0.013, 0.173), *p*=0.092 |
| MAXPR^f^ | 860 | 0.069 (0.004, 0.134), *p*=0.036 | -0.078 (-0.184, 0.029), *p*=0.152 |
| INTXSP | 860 | 0.270 (0.202, 0.338), *p*<0.001 | 0.192 (0.099, 0.285), *p*<0.001 |
| SRC | 860 | -0.115 (-0.179, -0.051), *p*<0.001 | -0.079 (-0.139, -0.018), *p*=0.011 |
| DRC | 860 | 0.170 (0.106, 0.235), *p*<0.001 | 0.142 (0.078, 0.207), *p*<0.001 |

Data are presented as β (95% confidence intervals). Model 1 includes age and sex. Model 2 includes total and HDL cholesterol, current smoking, presence of type 2 diabetes, brachial systolic BP, pharmacological anti-hypertensive treatment, study centre, body mass index, history of cardiovascular disease, estimated glomerular filtration rate and heart rate (above/below median) in addition to Model 1. ^a^The extent of collinearity was *r*=0.908 between MAXPR and aortic systolic BP, and variance inflation factor (VIF) for MAXPR was 6.987 and for aortic systolic BP was 7.352. ^b^The extent of collinearity was *r*=0.911 between INTXSP and aortic PP, and VIF for INTXSP was 6.258 and for aortic PP was 6.534. ^c^The extent of collinearity was *r*=0.824 between INTXSP and brachial PP, and VIF for INTXSP was 3.442 and for brachial PP was 3.391. ^d^The extent of collinearity was *r*=0.799 between MAXPR and brachial systolic BP, and VIF for MAXPR was 3.673 and for brachial systolic BP was 3.321. ^e^The extent of collinearity was *r*=0.801 between MAXPR and brachial systolic BP, and VIF for MAXPR was 3.346 and for brachial systolic BP was 3.541. ^f^The extent of collinearity was *r*=0.795 between MAXPR and brachial systolic BP, and VIF for MAXPR was 3.196 and for brachial systolic BP was 3.244. β, beta; INTPR, reservoir pressure integral; MAXPR, peak reservoir pressure; INTXSP, excess pressure integral; SRC, systolic rate constant; DRC, diastolic rate constant; BP, blood pressure; PP, pulse pressure; RAS, renin-angiotensin system; CI, confidence intervals.

**Supplemental Table S2.** Additional multivariable linear regression analyses to determine independent associations between reservoir-excess pressure parameters and NT-proBNP by replacing brachial diastolic BP and mean arterial pressure.

|  | **n** | **Model 1: β (95% CI)** | **Model 2: β (95% CI)** | |
| --- | --- | --- | --- | --- |
| ***Replacing brachial systolic BP with brachial diastolic BP*** | | | |  |
| INTPR | 868 | 0.134 (0.070, 0.198), *p*<0.001 | 0.213 (0.119, 0.307), *p*<0.001 | |
| MAXPR | 868 | 0.071 (0.006, 0.137), *p*=0.033 | 0.199 (0.096, 0.302), *p*<0.001 | |
| INTXSP | 868 | 0.271 (0.203, 0.339), *p*<0.001 | 0.232 (0.163, 0.301), *p*<0.001 | |
| SRC | 868 | -0127 (-0.196, -0.058), *p*<0.001 | -0.092 (-0.160, -0.024), *p*=0.008 | |
| DRC | 868 | 0.171 (0.107, 0.236), *p*<0.001 | 0.168 (0.104, 0.232), *p*<0.001 | |
| ***Replacing brachial systolic BP with mean arterial pressure*** | | | |  |
| INTPR | 868 | 0.134 (0.070, 0.198), *p*<0.001 | 0.136 (0.037, 0.234), *p*=0.007 | |
| MAXPR^g^ | 868 | 0.071 (0.006, 0.137), *p*=0.033 | 0.033 (-0.099, 0.165), *p*=0.625 | |
| INTXSP | 868 | 0.271 (0.203, 0.339), *p*<0.001 | 0.214 (0.137, 0.291), *p*<0.001 | |
| SRC | 868 | -0.127 (-0.16, -0.058), *p*<0.001 | -0.092 (-0.159, -0.025), *p*=0.007 | |
| DRC | 868 | 0.171 (0.107, 0.236), *p*<0.001 | 0.152 (0.088, 0.216), *p*<0.001 | |

Data are presented as β (95% confidence intervals). Model 1 includes age and sex. Model 2 includes total and HDL cholesterol, current smoking, presence of type 2 diabetes, brachial systolic BP, pharmacological anti-hypertensive treatment, study centre, body mass index, history of cardiovascular disease, estimated glomerular filtration rate and heart rate (above/below median) in addition to Model 1. ^g^The extent of collinearity was *r*=0.853 between MAXPR and mean arterial pressure, and variance inflation factor (VIF) for MAXPR was 4.701 and for mean arterial pressure was 4.573. β, beta; BP, blood pressure; INTPR, reservoir pressure integral; MAXPR, peak reservoir pressure; INTXSP, excess pressure integral; SRC, systolic rate constant; DRC, diastolic rate constant; CI, confidence intervals.

**Supplemental Table S3.** Additional multivariable linear regression analyses to determine independent associations between reservoir-excess pressure parameters, aortic parameters and NT-proBNP.

| **Reservoir-excess pressure parameter** | **β (95% CI)** | **Aortic parameter** | **β (95% CI)** |
| --- | --- | --- | --- |
| ***Adding reservoir-excess pressure parameter and aortic systolic BP in the same model*** | | | |
| INTPR | 0.056 (-0.051, 0.163), *p*=0.303 | Systolic BP | 0.159 (0.068, 0.250), *p*<0.001 |
| MAXPR^h^ | -0.287 (-0.448, -0.125), *p*<0.001 | Systolic BP | 0.459 (0.295, 0.622), *p*<0.001 |
| INTXSP | 0.175 (0.075, 0.275), *p*<0.001 | Systolic BP | 0.074 (-0.020, 0.168), *p*=0.124 |
| SRC | -0.075 (-0.143, -0.008), *p*=0.029 | Systolic BP | 0.193 (0.128, 0.259), *p*<0.001 |
| DRC | 0.136 (0.071, 0.201), *p*<0.001 | Systolic BP | 0.158 (0.091, 0.225), *p*<0.001 |
| ***Adding reservoir-excess pressure parameters and aortic PP in the same model*** | | | |
| INTPR | 0.077 (-0.011, 0.165), *p*=0.085 | PP | 0.200 (0.119, 0.282), *p*<0.001 |
| MAXPR | -0.026 (-0.113, 0.060), *p*=0.552 | PP | 0.255 (0.161, 0.350), *p*<0.001 |
| INTXSP^i^ | 0.121 (-0.034, 0.276), *p*=0.126 | PP | 0.126 (-0.032, 0.284), *p*=0.118 |
| SRC | -0.051 (-0.118, 0.016), *p*=0.138 | PP | 0.231 (0.160, 0.302), *p*<0.001 |
| DRC | 0.125 (0.059, 0.190), *p*<0.001 | PP | 0.197 (0.124, 0.270), *p*<0.001 |

Data are presented as β (95% confidence intervals). n= 852. Both reservoir-excess pressure parameters and aortic parameters were standardised for statistical analysis. The model includes age, sex, total and HDL cholesterol, current smoking, presence of type 2 diabetes, pharmacological anti-hypertensive treatment, study centre, body mass index, history of cardiovascular disease, estimated glomerular filtration rate and heart rate (above/below median). ^h^The extent of collinearity was *r*=0.909 between MAXPR and aortic systolic BP, and variance inflation factor (VIF) for MAXPR was 7.138 and for aortic systolic BP was 7.516. ^i^The extent of collinearity was *r*=0.917 between INTXSP and aortic pulse pressure, and VIF for INTXSP was 6.776 and for aortic pulse pressure was 7.007. β, beta; INTPR, reservoir pressure integral; MAXPR, peak reservoir pressure; INTXSP, excess pressure integral; SRC, systolic rate constant; DRC, diastolic rate constant; BP, blood pressure; PP, pulse pressure; CI, confidence intervals.

**Supplemental Table S4.** Additional multivariable linear regression analyses to determine independent associations between reservoir-excess pressure parameters and NT-proBNP by stratifying participants’ sex.

|  | **n** | **Model 1: β (95% CI)** | **Model 2: β (95% CI)** |
| --- | --- | --- | --- |
| ***Male*** | | | |
| INTPR | 549 | 0.122 (0.041, 0.204), *p*=0.003 | 0.062 (-0.053, 0.178), *p*=0.290 |
| MAXPR^j^ | 549 | 0.059 (-0.031, 0.149), *p*=0.200 | -0.120 (-0.266, 0.026), *p*=0.108 |
| INTXSP | 549 | 0.320 (0.228, 0.416), *p*<0.001 | 0.155 (0.027, 0.283), *p*=0.017 |
| SRC | 549 | -0.201 (-0.299, -0.103), *p*<0.001 | -0.090 (-0.185, 0.004), *p*=0.062 |
| DRC | 549 | 0.176 (0.087, 0.264), *p*<0.001 | 0.130 (0.043, 0.217), *p*=0.003 |
| ***Female*** | | | |
| INTPR | 319 | 0.155 (0.050, 0.259), *p*=0.004 | 0.125 (-0.032, 0.283), *p*=0.117 |
| MAXPR^k^ | 319 | 0.084 (-0.010, 0.179), *p*=0.079 | 0.006 (-0.152, 0.163), *p*=0.942 |
| INTXSP | 319 | 0.203 (0.103, 0.303), *p*<0.001 | 0.175 (0.037, 0.312), *p*=0.013 |
| SRC | 319 | -0.048 (-0.143, 0.047), *p*=0.321 | -0.041 (-0.130, 0.049), *p*=0.374 |
| DRC | 319 | 0.164 (0.071, 0.257), *p*<0.001 | 0.144 (0.051, 0.237), *p*=0.003 |

Data are presented as β (95% confidence intervals). Model 1 includes age. Model 2 includes total and HDL cholesterol, current smoking, presence of type 2 diabetes, brachial systolic BP, pharmacological anti-hypertensive treatment, study centre, body mass index, history of cardiovascular disease, estimated glomerular filtration rate and heart rate (above/below median) in addition to Model 1. ^j^The extent of collinearity was *r*=0.780 between MAXPR and brachial systolic BP, and variance inflation factor (VIF) for MAXPR was 3.143 and for brachial systolic BP was 3.109. ^k^The extent of collinearity was *r*=0.818 between MAXPR and brachial systolic BP, and VIF for MAXPR was 3.460 and for brachial systolic BP was 3.715. β, beta; INTPR, reservoir pressure integral; MAXPR, peak reservoir pressure; INTXSP, excess pressure integral; SRC, systolic rate constant; DRC, diastolic rate constant; PP, pulse pressure; RAS, renin-angiotensin system; CI, confidence intervals.

**Supplemental Table S5.** Additional multivariable linear regression analyses to determine independent associations between reservoir-excess pressure parameters and NT-proBNP by stratifying participants above/below 65 yrs of age.

|  | **n** | **Model 1: β (95% CI)** | **Model 2: β (95% CI)** |
| --- | --- | --- | --- |
| ***Below 65 yrs*** | | | |
| INTPR | 209 | 0.202 (0.065, 0.338), *p*=0.004 | 0.137 (-0.071, 0.345), *p*=0.197 |
| MAXPR^l^ | 209 | 0.185 (0.030, 0.340), *p*=0.019 | 0.012 (-0.244, 0.268), *p*=0.926 |
| INTXSP | 209 | 0.367 (0.193, 0.540), *p*<0.001 | 0.195 (-0.036, 0.426), *p*=0.017 |
| SRC | 209 | -0.045 (-0.167, 0.076), *p*=0.462 | -0.006 (-0.123, 0.110), *p*=0.913 |
| DRC | 209 | 0.219 (0.115, 0.323), *p*<0.001 | 0.177 (0.068, 0.286), *p*=0.002 |
| ***Above 65 yrs*** | | | |
| INTPR | 659 | 0.118 (0.045, 0.191), *p*=0.001 | 0.076 (-0.029, 0.181), *p*=0.157 |
| MAXPR^m^ | 659 | 0.047 (-0.026, 0.119), *p*=0.206 | -0.086 (-0.207, 0.034), *p*=0.160 |
| INTXSP | 659 | 0.255 (0.180, 0.329), *p*<0.001 | 0.187 (0.083, 0.291), *p*<0.001 |
| SRC | 659 | -0.168 (-0.253, -0.083), *p*<0.001 | -0.103 (-0.184, -0.022), *p*=0.013 |
| DRC | 659 | 0.145 (0.063, 0.228), *p*<0.001 | 0.118 (0.036, 0.200), *p*=0.005 |

Data are presented as β (95% confidence intervals). Model 1 includes age and sex. Model 2 includes total and HDL cholesterol, current smoking, presence of type 2 diabetes, brachial systolic BP, pharmacological anti-hypertensive treatment, study centre, body mass index, history of cardiovascular disease, estimated glomerular filtration rate and heart rate (above/below median) in addition to Model 1. ^l^The extent of collinearity was *r*=0.790 between MAXPR and brachial systolic BP, and variance inflation factor (VIF) for MAXPR was 3.239 and for brachial systolic BP was 3.256. ^m^The extent of collinearity was *r*=0.800 between MAXPR and brachial systolic BP, and VIF for MAXPR was 3.282 and for brachial systolic BP was 3.266. β, beta; INTPR, reservoir pressure integral; MAXPR, peak reservoir pressure; INTXSP, excess pressure integral; SRC, systolic rate constant; DRC, diastolic rate constant; CI, confidence intervals.

**Supplemental Table S6.** Additional multivariable linear regression analyses to determine independent associations between reservoir-excess pressure parameters and NT-proBNP by stratifying participants with/without the history of cardiovascular disease.

|  | **n** | **Model 1: β (95% CI)** | **Model 2: β (95% CI)** |
| --- | --- | --- | --- |
| ***Without CVD*** | | | |
| INTPR | 434 | 0.148 (0.066, 0.230), *p*<0.001 | 0.103 (-0.018, 0.224), *p*=0.095 |
| MAXPR^n^ | 434 | 0.143 (0.058, 0.228), *p*<0.001 | -0.001 (-0.148, 0.147), *p*=0.994 |
| INTXSP | 434 | 0.226 (0.127, 0.326), *p*<0.001 | 0.146 (0.007, 0.285), *p*=0.040 |
| SRC | 434 | -0.032 (-0.114, 0.050), *p*=0.441 | -0.039 (-0.120, 0.041), *p*=0.339 |
| DRC | 434 | 0.117 (0.032, 0.202), *p*=0.007 | 0.090 (0.000, 0.180), *p*=0.051 |
| ***With CVD*** | | | |
| INTPR | 434 | 0.151 (0.062, 0.239), *p*<0.001 | 0.063 (-0.081, 0.206), *p*=0.391 |
| MAXPR^o^ | 434 | 0.090 (-0.002, 0.182), *p*<0.001 | 0.030 (0.015, 0.045), *p*=0.131 |
| INTXSP | 434 | 0.227 (0.136, 0.317), *p*<0.001 | 0.206 (0.075, 0.337), *p*=0.002 |
| SRC | 434 | -0.138 (-0.249, -0.027), *p*=0.015 | -0.128 (-0.237, -0.018), *p*=0.022 |
| DRC | 434 | 0.188 (0.100, 0.277), *p*<0.001 | 0.182 (0.090, 0.275), *p*<0.001 |

Data are presented as β (95% confidence intervals). Model 1 includes age and sex. Model 2 includes total and HDL cholesterol, current smoking, presence of type 2 diabetes, brachial systolic BP, pharmacological anti-hypertensive treatment, study centre, body mass index, estimated glomerular filtration rate and heart rate (above/below median) in addition to Model 1. ^n^The extent of collinearity was *r*=0.797 between MAXPR and brachial systolic BP, and variance inflation factor (VIF) for MAXPR was 3.197 and for brachial systolic BP was 3.262. ^o^The extent of collinearity was *r*=0.806 between MAXPR and brachial systolic BP, and VIF for MAXPR was 3.268 and for brachial systolic BP was 3.428. β, beta; CVD, cardiovascular disease; INTPR, reservoir pressure integral; MAXPR, peak reservoir pressure; INTXSP, excess pressure integral; SRC, systolic rate constant; DRC, diastolic rate constant; CI, confidence intervals.
